# Unmasking a Case of Asymptomatic Charcot-Marie-Tooth Disease (CMT1A) With Vincristine

**DOI:** 10.1177/2324709618758349

**Published:** 2018-02-26

**Authors:** Roopam Jariwal, Basel Shoua, Katayoun Sabetian, Piruthiviraj Natarajan, Everardo Cobos

**Affiliations:** 1 Kern Medical Center, Bakersfield, CA, USA

**Keywords:** vincristine, peripheral myelin protein (PMP) 22 gene, hereditary motor and sensory neuropathy, Charcot-Marie-Tooth disease (CMT1A), electromyography, nerve conduction study, neurotoxic, chromosome 17p11.2

## Abstract

Charcot-Marie-Tooth (CMT) disease is a hereditary demyelinating disease of the peripheral nervous system that results in sensory and motor dysfunction. CMT includes a spectrum of diseases with different types of mutations in the genes encoding myelin protein, resulting in a variety of dysfunctions in its life cycle. In CMT subtype 1A there is duplication mutation of peripheral myelin protein 22 gene on chromosome 17. Incomplete penetrance, gene-dosage effect, and variable expressivity can attribute to the asymptomatic nature of the disease in some subset of patients. Vincristine administration is contraindicated in patients who are alrea\dy diagnosed with CMT disease. We report a case of asymptomatic CMT disease unmasked only by the neurotoxic effects of vincristine. Genetic testing for a patient with a preexisting family background of inherited diseases before starting vincristine therapy can potentially prevent a disability.

## Introduction

Charcot-Marie-Tooth (CMT) disease is a hereditary motor and sensory neuropathy characterized by peripheral nerve dysfunction. Prevalence of CMT disease is 1/2500 of the general population, and CMT 1A disease is the most common type.^1^ They are classified (CMT 1-7) based on different abnormalities and dysfunction of myelin structure, development, and function.^2^ Except for the CMT type 2 neuropathy (axonal non-demyelinating), most of the other CMT disease types show some form of demyelination and, in addition, are associated with individual clinical characteristics depending on the inherited mutations. CMT1A disease, a subtype of CMT1, constitute around 60% of all CMT disease. The duplication mutation amounts to 90% of CMT1A disease, and the remaining 10% are point mutations.^3^ Patients with preexisting neuropathic conditions are at potential risk for significant neurological damage with vincristine administration.^4-6^

## Case Presentation

**Figure 1.**
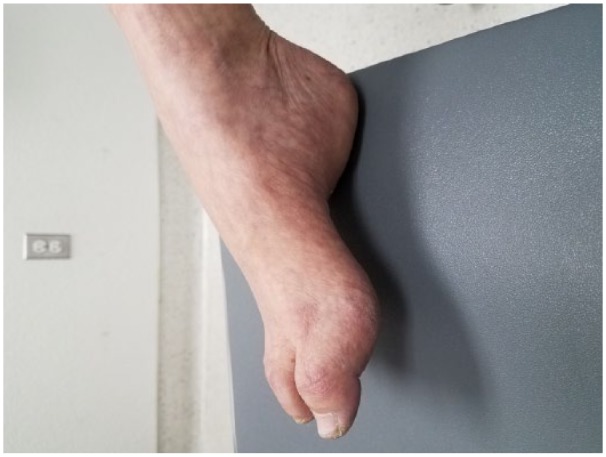
Right foot: pes cavus and hammertoes.

A 56-year-old Hispanic woman presented with severe abdominal pain for the past 2 weeks. She had intermittent low-grade abdominal pain for the past 4 months. During her recent visit to Mexico a few months ago, she was diagnosed with splenic enlargement and had her spleen removed. On evaluation at our facility, the lymph node biopsy revealed diffuse large B-cell lymphoma for which she received R-CHOP chemotherapy (cyclophosphamide, doxorubicin, vincristine, prednisone, and rituximab). Within 4 days of the first cycle of chemotherapy, she had mild weakness, numbness at the tip of her fingers and toes, and it extended to all 4 limbs in 10 days of the second cycle. In the next few days her symptoms worsened, and she was unable to perform her activities of daily life. On examination, light touch and pain (pinprick) in all 4 limbs distal to both elbows and knees were decreased with motor weakness grade 3/5 in all 4 limbs. After the development of the polyneuropathic symptoms during the review, she was diagnosed with high arched feet (Figure 1). Her siblings and their father had high arched feet and hammertoes. Everyone in the family were asymptomatic and did not suffer any disability as a result of the feet deformities.

Nerve conduction study of bilateral upper and lower limbs revealed absent action potentials in bilateral median, ulnar, peroneal, right tibial motor, and sural sensory nerves with the exception of left tibial motor nerve, which showed severe axonal and severe demyelinating polyneuropathy. Electromyography showed severe active denervation and no recruitment, or severely reduced (neurogenic) recruitment of the motor units in forearm, hands, proximal, and distal legs. Proximal arm muscles were spared. The nerve conduction study and electromyography were consistent with severe diffuse sensory motor axonal and demyelinating acute superimposed on chronic polyneuropathy. Genetic testing showed a pathogenic variant and duplication of the entire peripheral myelin protein 22 (PMP22) gene. The PMP22 gene duplication was consistent with the diagnosis of CMT subtype IA with autosomal dominant manifestation. Vincristine was discontinued when clinical diagnosis of CMT disease was suspected and received rituximab. The motor symptoms gradually improved, and the latest follow-up after 6 months showed some residual sensory neuropathy.

## Discussion

CMT is a common heritable peripheral neuropathy exhibiting acute severe neurotoxicity after vincristine administration.^4-7^ CMT1A is an autosomal dominant disease resulting from the duplication of PMP22 gene of chromosome 17p11.2.^8^ The duplication mutation is classified as CMT1A associated with increased expression of PMP. An increase in PMP affects multiple cell signaling pathways and attributes to dysfunction of transcriptional factors.^9^ The development of symptoms and their exacerbation are directly related to the pattern of phenotypic expression of the genes referred to as gene-dosage effect.^10^ A deletion of the same gene (PMP22) results in hereditary neuropathy with liability to pressure palsy exhibiting a different type of neuropathy.^11^ The function of the PMP22 gene is likely to modulate myelin production, differentiation, and death. CMT1 and its subtypes usually present with weakness in distal muscles of the leg with loss of deep tendon reflexes, distal calf atrophy, pes cavus, and hammertoe, and they are associated with late findings of atrophy of intrinsic muscles of the hand and palpable enlargement of peripheral nerves. CMT1A disease usually presents in the first 2 decades of life, whereas our patient was asymptomatic until 58 years of age except for the pes cavus and hammertoe. Incomplete penetrance could be attributed to the unique presentation, which was unmasked only by vincristine administration.^12^ In spite of the standard dosing, aggressive neurotoxic symptom development within a period of days could suggest an increased sensitivity to vincristine in a preexisting CMT1A disease.^13^ A late-onset CMT disease were reported previously with early growth response 2 (EGR2), myelin protein zero (MPZ) mutations of other CMT types but never reported in the PMP22 gene.^14^ Vincristine suppresses the microtubules during cell cycle, blocking the metaphase stage of mitosis. The cell unable to complete the mitosis has to undergo apoptosis. Avoiding neurotoxic drugs in a diagnosed case of CMT disease could prevent a decompensation. It is also important to evaluate other chemotherapeutic drugs that are notorious for neuropathy like paclitaxel, cisplatin, interferon, and podophyllotoxins and their effects on preexisting neuropathic conditions.^15^ The genetic testing for CMT or any neurological disorders are currently not a standard of care before administration of vincristine chemotherapy. In this outset a detailed family history to rule out hereditary neuropathic diseases and a possible genetic testing on suspected patients could possibly prevent a potential permanent disability.

## Conclusion

Prior to starting chemotherapeutic agents notorious for causing neuropathy, we recommend examination of patient’s feet for deformities such as pes cavus and hammertoes. If present, the patient should be referred for neurological evaluation and a further nerve conduction study and electromyography to look for underlying hereditary or acquired peripheral neuropathy prior to initiating the drug.
